# Cognitive and Behavioral Effects of Levetiracetam in Juvenile Rats

**DOI:** 10.1002/prp2.70226

**Published:** 2026-03-05

**Authors:** Sheetal D. Ullal, Ashima G. Thomas, Sahana Devadasa Acharya, Vandana Blossom, Rajeshwari Shastry

**Affiliations:** ^1^ Department of Pharmacology Kasturba Medical College Mangalore, Manipal Academy of Higher Education Manipal India; ^2^ Department of Anaesthesia Jubliee Mission Medical College Thrissur India; ^3^ Department of Anatomy Kasturba Medical College Mangalore, Manipal Academy of Higher Education Manipal India

**Keywords:** behavioral effects, cognition, juvenile rats, levetiracetam

## Abstract

Epilepsy is a common neurologic disorder; its incidence in childhood is 2.5 per 1000 children. Levetiracetam (LEV) is an antiepileptic drug with a broad spectrum of action used in localized and generalized epilepsies in both adults and children. Studies have shown adverse behavioral effects, such as somnolence, agitation, and nonpsychotic mood disorders in short‐term studies. The paucity of properly designed long‐term studies to determine the impact of LEV treatment in children encouraged us to analyze the long‐term effect of LEV in juvenile Wistar rats. The objective was to study the impact of LEV on cognition and behavior in juvenile rats. Six Wistar rats each were randomly allocated to: Group 1: Control—Received 0.9% NaCl at a dose of 10 mL/kg body weight; 2: Received LEV 200 mg/kg dose; 3: Received LEV 400 mg/kg dose. All drugs were administered once daily orally for 21 days, starting from the third postnatal week to the sixth postnatal week. Compared with the control group, both the 200 mg/kg and 400 mg/kg LEV‐treated groups subjected to behavioral paradigms did not decrease learning and memory. Histopathological examination revealed a significant decrease in neuronal density in the dentate gyrus, hippocampus, and frontal cortex in both the 200 mg/kg and 400 mg/kg LEV‐treated groups compared with the control group. Although LEV remains a valuable antiepileptic agent, this study highlights the importance of cautious dosing and vigilant monitoring, especially in the long‐term use of LEV, as histopathological findings revealed a dose‐dependent decrease in the neuronal count.

**Abbreviations**
ANOVA: Analysis of Variance
CA: Cornu ammonis
CA1: CA2
CA3: CA4: Hippocampal subregions
DG: Dentate gyrus
FC: Frontal cortex
HIF‐1α: Hypoxia‐inducible factor 1‐alpha
LEV: Levetiracetam
RTS: Retention score
SD: Standard deviation
SPSS: Statistical Package for the Social Sciences
SV2A: Synaptic Vesicle Glycoprotein 2A
TRC: Time to reach the reward chamber

## 1 Introduction

Epilepsy is a prevalent neurological condition, with a pooled incidence of 2.5 per 1000 children [1]. Levetiracetam (LEV), a newer‐generation antiepileptic drug, is approved for use in both adults and children and ranks among the most prescribed medications for epilepsy. It is effective as monotherapy in managing both focal and generalized epilepsy in pediatric populations [2]. LEV exerts its action by modulating synaptic neurotransmitter release through binding to the synaptic vesicle protein SV2A in the brain.

Pharmacokinetically, LEV exhibits a linear profile, is rapidly and completely absorbed, has high oral bioavailability, shows minimal plasma protein binding, does not induce hepatic enzymes, and displays minimal drug interactions. It is primarily eliminated via renal clearance, with partial metabolism occurring in the blood [3].

Experimental studies have shown dose‐dependent impairment in midline closure during early embryonic development in chickens [4]. Although current research on prenatal LEV exposure does not reveal a significant dose‐related impact on neurodevelopment, further studies are necessary to establish its safety profile [5]. In children, behavioral disturbances and somnolence are among the most frequently reported adverse effects [6, 7]. A randomized, placebo‐controlled crossover study conducted in Florida revealed LEV to be well tolerated in elderly individuals, with no detrimental effects on cognition or behavior. Given its pharmacokinetic advantages, LEV is considered a suitable anticonvulsant for older adults [8].

Owing to the limited data on the long‐term effects of LEV in pediatric populations, we undertook a preclinical study to evaluate its impact following prolonged administration in juvenile Wistar rats. The objective of this study was to assess the effects of LEV on cognitive function and learned helplessness behavior in juvenile rats.

## 2 Materials and Methods

### 2.1 Animals and Ethics Approval

In‐house‐bred albino Wistar rats of either sex were used for the study, with six animals in each group. The animals were provided ad libitum access to food and water. They were housed under controlled environmental conditions, including a 12‐h light/dark cycle, a temperature maintained at 22°C ± 3°C, a relative humidity of approximately 50% ± 10%, and a pathogen‐free environment.

The rats were housed in polypropylene cages with paddy husk used as bedding material. All experimental procedures were approved by the Institutional Animal Ethics Committee before commencement and were conducted under the guidelines laid down by the Committee for Control and Supervision of Experiments on Animals (CCSEA).

### 2.2 Drugs and Chemicals

LEV tablets of dose 750 mg (Sun Pharma Laboratories) were purchased for the study. The tablets were crushed to make a powder, suspended in 2% Gum acacia to be given orally.

### 2.3 Drug Administration Protocol

Group 1 (Control): Rats in the control group received 2% gum acacia at a dose of 1 mL/kg body weight once daily via oral gavage for 3 weeks (21 days).

Group 2: Rats in this group received LEV at a dose of 200 mg/kg orally once daily via oral gavage for 3 weeks (21 days).

Group 3: Rats in this group received LEV at a dose of 400 mg/kg orally once daily via oral gavage for 3 weeks (21 days).

All the treatments, whether drug or vehicle, were administered orally once per day over 3 weeks, from the third postnatal week to the sixth postnatal week. This schedule was chosen because, by the third postnatal week, the first adult‐like neurons begin to appear in the hippocampal dentate gyrus of rats, and active synaptogenesis and neuronal development are underway in the brain [9].

### 2.4 Cognitive Function Evaluation

#### 2.4.1 Condition Avoidance Test

The conditioned avoidance test was employed to assess learning and memory functions in rats via a shuttle box apparatus. The shuttle box consisted of a closed wooden enclosure with shutters at the front and a grid floor divided into two compartments by a median grid. Each compartment was connected to an independent electric circuit, and a buzzer was installed to deliver auditory cues. Each rat was allowed to explore the shuttle box for 5 min. Later, a discriminative stimulus (buzzer sound) was given for a 10‐s interval. During this stimulus period, the rat could avoid electric shock by crossing to the opposite compartment. If the rat failed to respond during the stimulus, it received a 2.5 mA shock for a maximum of 10 s, during which it could escape by crossing the median grid [10].

The avoidance response was defined as a single crossing over the median grid during the stimulus period. Each rat underwent three trials per day for three consecutive days. Under normal conditions, the number of shock avoidances typically increases from Day 1 to Day 3, indicating learning progression. The mean score across all 3 days was calculated to represent the rat's learning performance.

To assess memory retention, each rat was retested 24 h after the final trial. The retention score (RTS) was calculated via the following formula:

$$\mathrm{RTS}=\left(\text{Mean of retest score}\;X\;\text{Mean scoring of days}\;1--3\;\text{of testing}\right)/\left(\text{Mean score during}\;\mathrm{day}\;3\;\text{of the testing}\right)$$

A decrease in the RTS was considered an indication of memory impairment.

#### 2.4.2 Spatial and Working Memory Assessment: Hebb–Williams Maze

The Hebb–Williams Maze is an incentive‐based exteroceptive behavioral model used to evaluate spatial and working memory in rats. The apparatus comprises three compartments:

1. Start Box (Animal Chamber): The rat is initially placed here. A guillotine‐style removable door allows access to the next compartment.
2. Middle Chamber (Exploratory Area): Upon entry, the guillotine door is closed to prevent re‐entry into the start box. The rat explores this area and navigates toward the reward chamber.
3. Reward chamber: Located at the far end of the maze, this chamber contains a food reward.

An integrated electrical system detects the rat's movement between compartments, enabling automated recording of the time to reach the reward chamber (TRC) using a digital timer. Each rat was allowed an additional 20 s of free exploration with all doors open before being returned to its home cage [11].

Overnight‐fasted rats were used to increase motivation. Each rat underwent three sessions of 5 min (300 s) each. If a rat failed to reach the reward chamber within 5 min, the TRC was recorded as 300 s. The mean TRC across the three sessions was calculated for each animal.

An increase in TRC compared with that of control animals was interpreted as an indication of learning or memory impairment.

### 2.5 Assessment of Learned Helplessness Behavior

#### 2.5.1 Forced Swim Test

The forced swim test, as described by Porsolt et al., was used to evaluate depressant‐like behavior and potential learned helplessness in rats. The apparatus consisted of a transparent, white plastic tub measuring 37 cm in height and 30 cm in diameter, filled with tap water at room temperature to a depth of 25 cm. The water level was maintained such that the rat's tail did not touch the bottom of the tub, ensuring free movement [12].

The test was conducted during the first half of the day to minimize circadian variability. Each rat was placed individually in the tub, and a stopwatch was started immediately. The duration of immobility was recorded during the last 4 min of the 6‐min test period.

A rat was considered immobile when it floated passively, making only minimal movements necessary to keep its head above the water surface. The water was changed after each test to maintain hygiene and consistency.

An increased duration of immobility was interpreted as an indicator of behavioral despair, suggesting the potential of the administered compound to induce depressant‐like effects or learned helplessness.

### 2.6 Neuronal Density Assessment: Nissl Staining

To assess neuronal density, the animals were deeply anesthetized with ketamine and secured on a dissection board. The thoracic cavity was opened to expose the heart, and transcardial perfusion was performed via the left ventricle using 100–150 mL of 0.9% saline at a flow rate of 1 mL/min, followed by 10% formalin at a similar rate.

Postperfusion, the animals were decapitated, and the brains were carefully extracted and immersed in 10% formalin for 48 h for postfixation. Paraffin embedding was performed via an embedding bath, and coronal sections 5–6 μm thick were cut through the hippocampus using a rotary microtome.

A total of 25 serial sections per animal were mounted on air‐dried gelatinized slides and stained with cresyl violet. The staining solution was prepared by dissolving 100 mg of cresyl violet in 100 mL of distilled water, with 0.5 mL of 10% acetic acid added to maintain a pH of 3.5–3.8. The solution was warmed and filtered before use.

Quantitative analysis was performed under 20X magnification using a Nikon H600L trinocular microscope. Specific regions were selected for neuronal counting: dentate gyrus: 150 μm^2^ area; frontal cortex: 250 μm^2^ area; and cornu ammonis (CA1, CA2, CA3, CA4): 300 μm linear segment per region. Neuronal quantification was carried out using NIS Elements BR software (version 4.30) [13].

### 2.7 Statistical Analysis

Data following a normal distribution were expressed as the mean ± standard deviation (SD), and statistical analysis was performed via one‐way ANOVA followed by Tukey's post hoc test. A value of *p* < 0.05 was considered statistically significant.

## 3 Results

### 3.1 Condition Avoidance Test

The retention trial was conducted 24 h after the completion of the three‐day acquisition trial. During the discriminative stimulus period, the rat avoided shock by jumping to the other compartment. Such shock avoidance responses were observed during both the acquisition and retention trials. The mean scores for the acquisition and retention trials across different groups are presented in Table 1.

**TABLE 1:** Effects of levetiracetam treatment on neonatal rats in the condition avoidance test.

Group	Mean ± SD shock avoidance/day in acquisition trial	Mean ± SD shock avoidance during retest	Mean ± SD retention score (RTS)
Control	1 ± 0.24	1.5 ± 0.5	0.83 ± 0.78
LEV 200 mg/kg	1 ± 0.24	1.83 ± 0.48	0.75 ± 0.58
LEV 400 mg/kg	1.23 ± 0.23	2 ± 0.85	1.07 ± 0.7
*p* value	0.649	0.755	0.702

There was no statistically significant difference in the shock avoidance/day in the acquisition trial (3 days of the acquisition trial) among the normal control group, the LEV 200 mg/kg group, and the LEV 400 mg/kg group. Similarly, no significant difference was observed in the shock avoidance during the retention trial and in the RTS among the three groups.

### 3.2 Hebb‐William Maze Test

Twenty‐four hours after training, the time taken to reach the TRC was recorded. The results, expressed as the mean ± SD, were as follows: normal control group, 31.33 ± 14.3 s; LEV 200 mg/kg group, 29.5 ± 8.96 s; and LEV 400 mg/kg group, 32.33 ± 12.49 s. Statistical analysis revealed no significant difference in the TRC among the three groups (Table 2).

**TABLE 2:** Effects of levetiracetam treatment in neonatal rats via the Hebb–William Maze.

Group	Time taken to reach the reward chamber (TRC) in seconds Mean ± SD
Control	31.33 ± 14.3
LEV 200 mg/kg	29.5 ± 8.96
LEV 400 mg/kg	32.33 ± 12.49
*p* value	0.920

### 3.3 Forced Swim Test

The forced swim test was used to assess behavioral despair, with the duration of immobility serving as an indicator of learned helplessness. The mean immobility durations (in seconds) for the three experimental groups are presented in Table 3. Compared with normal control rats, rats treated with LEV at doses of 200 mg/kg and 400 mg/kg presented increased immobility durations. However, these differences were not statistically significant, suggesting that levetiracetam at the tested doses did not markedly influence depressive‐like behavior in this model.

**TABLE 3:** Duration of immobility in the forced swim test.

Group	Duration of immobility in seconds Mean ± SD
Control	117.5 ± 29.72
LEV 200 mg/kg	146.67 ± 26.7
LEV 400 mg/kg	142 ± 16.38
*p* value	0.128

### 3.4 Histopathological Observations

**FIGURE 1:**
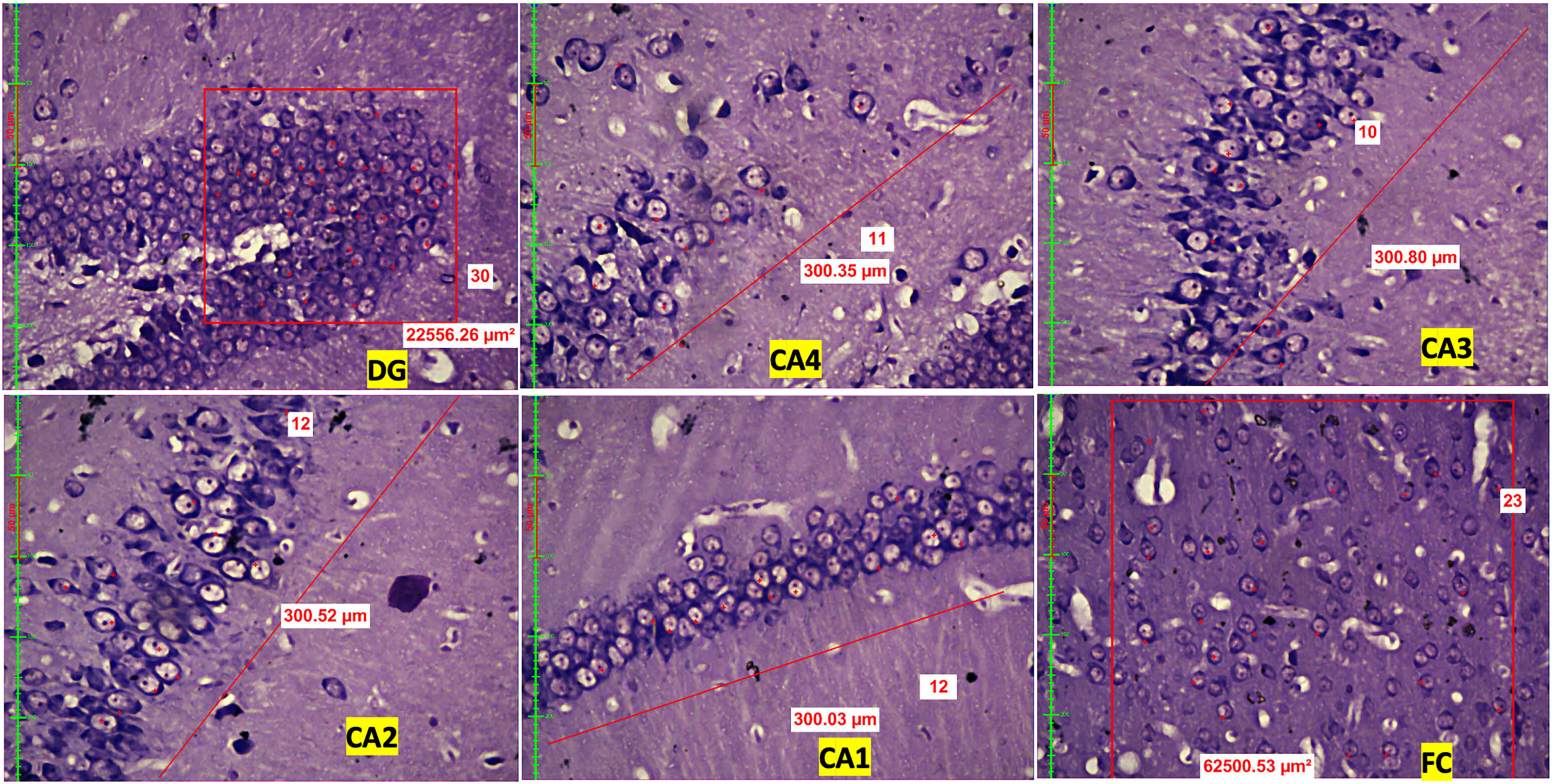
Photomicrographs of the Dentate Gyrus (DG), Cornu Ammonis (CA1–CA4) regions of the hippocampus, and frontal cortex (FC) in the normal control group, highlighting viable neurons with observable vacuolization.

**FIGURE 2:**
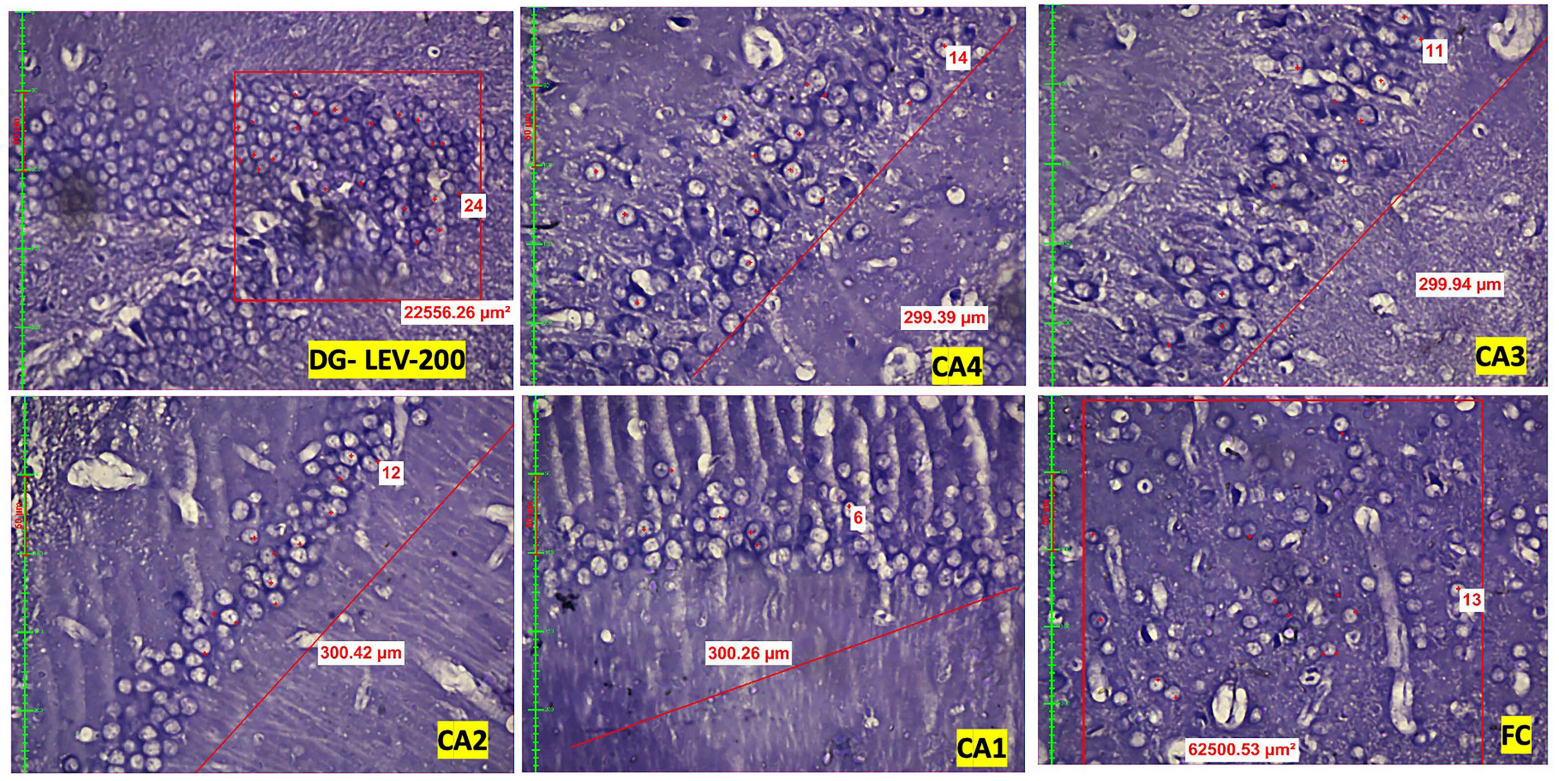
Photomicrographs of the Dentate Gyrus (DG), Cornu Ammonis (CA1–CA4) regions of the hippocampus, and frontal cortex (FC) in the LEV 200 mg/kg group, highlighting viable neurons with observable vacuolization.

**FIGURE 3:**
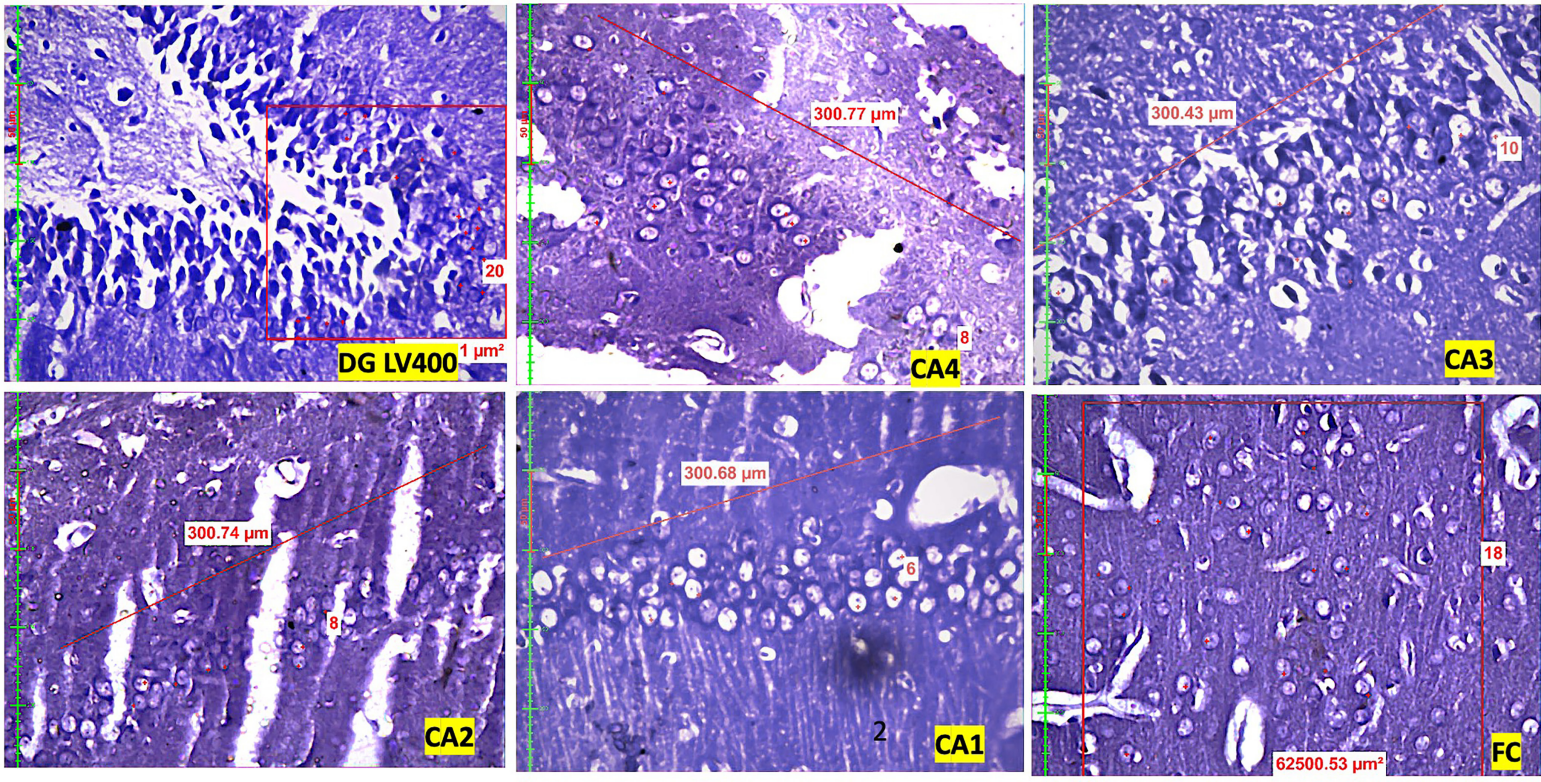
Photomicrographs of the Dentate Gyrus (DG), Cornu Ammonis (CA1–CA4) regions of the hippocampus, and frontal cortex (FC) in the LEV 400 mg/kg group, highlighting viable neurons with observable vacuolization.

**FIGURE 4:**
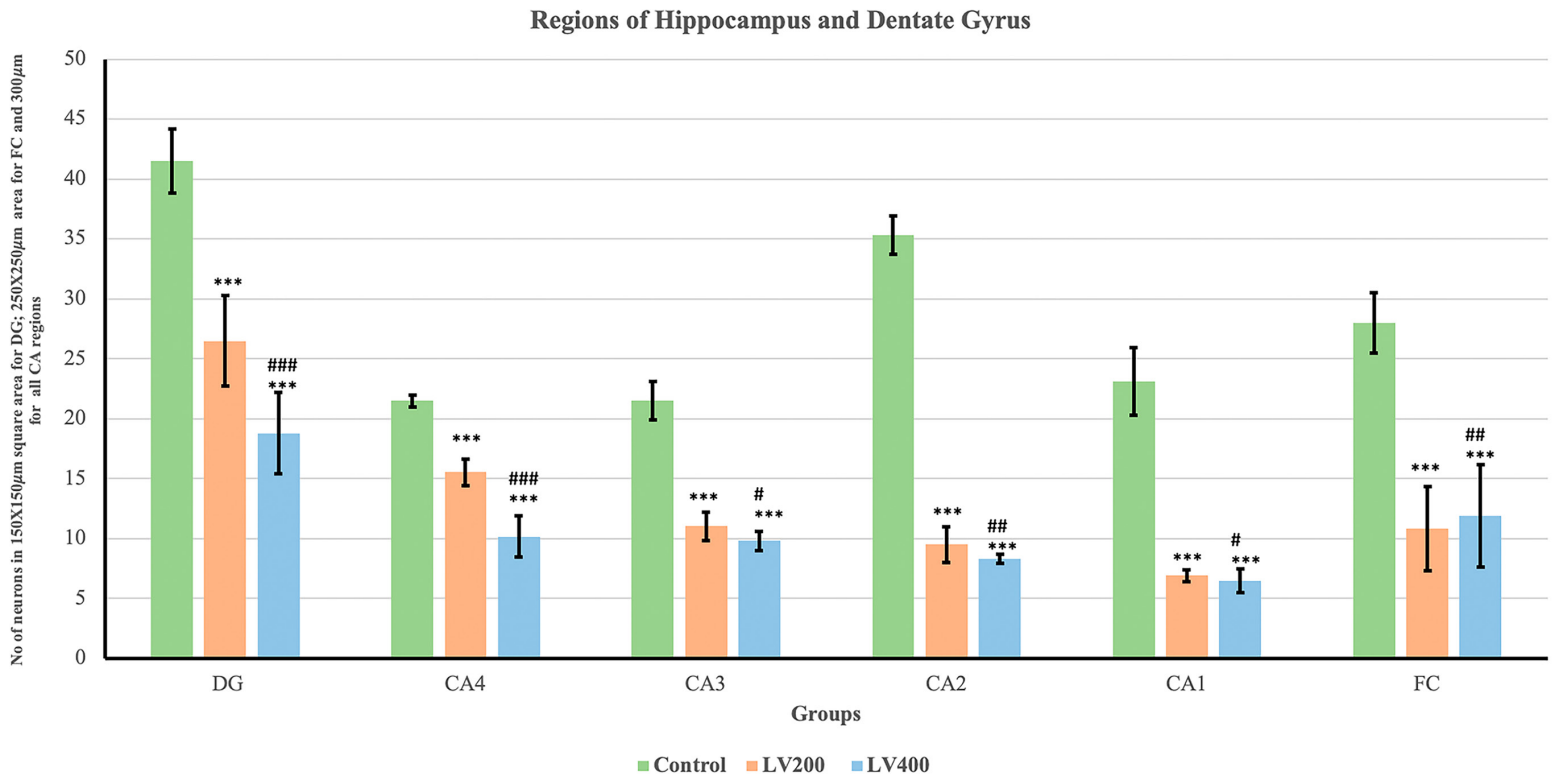
Comparison of the number of viable neurons in the dentate gyrus (DG), regions of the hippocampus (CA1‐CA4), and frontal cortex (FC) across groups (*n* = 6 for each group). ***Control vs. LEV200 mg/kg *p* < 0.001; ^###^LEV 200 mg/kg vs. LEV 400 mg/kg *p* < 0.001, ^##^ *p* < 0.01, ^#^ *p* < 0.05. One‐way ANOVA followed by Tukey's post hoc test was used; the error bars represent the standard deviation.

Compared with those in the control group, quantitative assessment of neuronal density revealed a significant dose‐dependent reduction in the number of neurons across the dentate gyrus (DG), hippocampal subregions (CA1, CA2, CA3, CA4), and frontal cortex (FC) in both the LEV 200 and the LEV 400 mg/kg groups (Figure 1, Figure 2, Figure 3). In the DG, the control group presented a mean neuronal count of 41.5 ± 2.7, which decreased to 26.5 ± 3.6 in the LEV 200 mg/kg group and further decreased to 18.8 ± 3.9 in the LEV400 mg/kg group (*p* < 0.001). In the CA4 region, the neuronal count decreased from 21.5 ± 0.5 in the control group to 15.5 ± 1.1 in the LEV 200 g and 10.2 ± 1.7 in the LEV 400 mg/kg group (*p* < 0.001). The CA3 region was reduced from 21.5 ± 1.6 in the control group to 11.2 ± 1.2 in the LEV 200 and 9.8 ± 0.8 in the LEV 400 mg/kg group (*p* < 0.001). In CA2, the number of viable neurons decreased markedly from 35.3 ± 1.6 in the control group to 9.5 ± 1.5 in the LEV 200 and 8.3 ± 0.4 in the LEV 400 mg/kg group (*p* < 0.001). Similarly, CA1 neuronal counts decreased from 23.1 ± 2.0 in the control group to 6.9 ± 0.5 in the LEV 200 and 6.5 ± 1 in the LEV 400 mg/kg group (*p* < 0.001). FC also exhibited reduced neuronal density, decreasing from 28 ± 2.5 in the control group to 10.8 ± 3.5 in the LEV 200 and 11.9 ± 4.3 in the LEV 400 mg/kg group (*p* < 0.001) (Figure 4). When either LEV 200 or LEV 400 mg/kg was compared with the control group, high statistical significance was observed across all regions, indicating altered neuronal viability. The reduction in the number of viable neurons highlights the neurotoxic effects of LEV.

## 4 Discussion

This study investigated the neurobehavioral and histopathological effects of LEV at doses of 200 mg/kg and 400 mg/kg in juvenile rats exposed to LEV for 3 weeks. The two doses administered in this study were within the therapeutic dose range for children. While behavioral assessments, including the condition avoidance test, Hebb–William's maze, and forced swim test, did not reveal statistically significant differences between the LEV‐treated and control groups, histopathological analysis demonstrated a clear dose‐dependent reduction in neuronal density across the dentate gyrus (DG), hippocampal subfields (CA1–CA4), and frontal cortex (FC).

In a prospective, nonrandomized study on neonatal seizures, LEV demonstrated both efficacy and safety [14]. However, the study has limitations due to its small sample size and nonrandomized design, and it did not assess long‐term effects on memory.

Conversely, a study by the Department of Pediatric Neurophysiology and Rehabilitation at the University of Poland reported that LEV did not exhibit neuroprotective effects on hippocampal neurons following heat‐induced injury in an in vitro hyperthermia model. Furthermore, at higher concentrations, LEV was found to intensify aponecrosis in cultured hippocampal neurons [15].

A systematic review revealed that behavioral disturbances and somnolence were common adverse effects of LEV in pediatric patients, often leading to discontinuation of treatment. These side effects were more prevalent in children receiving polytherapy than in those receiving LEV monotherapy [7].

Another study concluded that LEV does not promote apoptosis in the developing brains of neonatal mice or interfere with the neuroprotective upregulation of hypoxia‐inducible factor 1‐alpha (HIF‐1α) [16].

The absence of significant behavioral changes despite marked neuronal loss suggests a possible functional compensation or threshold effect, where behavioral deficits may only manifest beyond a certain level of neuronal damage. Alternatively, the behavioral paradigms used may lack the sensitivity to detect subtle cognitive or affective changes, especially in short‐term studies.

These histopathological findings raise concerns about the neurotoxic potential of LEV, particularly in the therapeutic dose range. Although LEV is widely used for seizure control and is generally considered safe, recent clinical studies have reported adverse behavioral effects, including agitation, depression, and cognitive impairment, in up to 46% of neurocritical care patients [17]. These effects are often underrecognized or misattributed to underlying neurological conditions [18]. Given the roles of the hippocampus and frontal cortex in memory and executive function, the observed neuronal loss may have implications for long‐term cognitive health, especially in vulnerable populations such as children, elderly patients, and those with preexisting neuropsychiatric conditions. This underscores the need for dose optimization, therapeutic drug monitoring, and regular neuropsychological assessments during LEV therapy.

The advantage of this study was the multimodal assessment, which included both behavioral and histopathological evaluations and provided a holistic view of LEV effects. The study clearly demonstrated a dose‐dependent pattern of neuronal loss, strengthening causal inference. Detailed quantification across hippocampal subfields and cortical regions enhances anatomical specificity.

The limitations of the study were the lack of functional correlation despite significant neuronal loss and the lack of corresponding deficits in the behavioral tests, possibly due to short observation periods or insensitive paradigms. Species differences, such as rodent models, may not fully replicate human neurophysiology, limiting translational applicability [19]. The study does not address whether neuronal loss is reversible or progressive over time. The effects of LEV in polytherapy contexts or in disease models (e.g., epilepsy and Alzheimer's disease) have not been explored.

## 5 Conclusion

While LEV remains a valuable antiepileptic agent, this study highlights the importance of cautious dosing and vigilant monitoring, especially for long‐term use. The disconnect between behavioral and histopathological findings necessitates more sensitive behavioral assays and longitudinal studies to understand the clinical relevance of LEV‐induced neuronal changes fully.

## Author Contributions

**Sheetal D. Ullal:** conceptualization, methodology, supervision. **Ashima G. Thomas:** data curation, writing – original draft preparation. **Sahana Devadasa Acharya:** conceptualization, methodology, writing – reviewing and editing, visualization. **Vandana Blossom:** data curation, data analysis. **Rajeshwari S:** supervision, writing – reviewing and editing.

## Funding

The authors have nothing to report.

## Conflicts of Interest

The authors declare no conflicts of interest.

## Data Availability

The data that support the findings of this study will be made available from the corresponding author upon reasonable request.
